# Novel Sustainable Composites Based on Poly(hydroxybutyrate-co-hydroxyvalerate) and Seagrass Beach-CAST Fibers: Performance and Degradability in Marine Environments

**DOI:** 10.3390/ma11050772

**Published:** 2018-05-11

**Authors:** Maurizia Seggiani, Patrizia Cinelli, Elena Balestri, Norma Mallegni, Eleonora Stefanelli, Alessia Rossi, Claudio Lardicci, Andrea Lazzeri

**Affiliations:** 1 Department of Civil and Industrial Engineering, University of Pisa, Largo Lucio Lazzarino 1, Pisa 56126, Italy; norma.mallegni@gmail.com (N.M.); eleonora.stefanelli@ing.unipi.it (E.S.); andrea.lazzeri@unipi.it (A.L.); 2 Department of Biology, University of Pisa, Via Derna 1, Pisa 56126, Italy; arossi.bio@hotmail.it (A.R.); claudio.lardicci@unipi.it (C.L.)

**Keywords:** poly(hydroxybutyrate-co-hydroxyvalerate), PHBV, *Posidonia oceanica*, biocomposites, biodegradability, degradation

## Abstract

In order to produce sustainable, bio-based and highly biodegradable materials, composites based on poly(hydroxybutyrate-co-hydroxyvalerate) (PHBV) and fibers of *Posidonia oceanica* (PO), a dominant Mediterranean seagrass, were produced by simple melt mixing and characterized in terms of thermal stability, morphology and rheological/mechanical properties. In view of their potential application in marine environments, degradation of the developed composites was evaluated under simulated and real marine environmental conditions for 1 year. Using 10 wt % of acetyl tributyl citrate (ATBC) as a plasticizer, smooth processing was achieved for up to 30 wt % of PO fibers, despite the reduction of the melt fluidity observed with increasing fiber loading. The tensile modulus slightly increased (from 2 to 2.4 GPa) while the tensile strength and the elongation decreased (from 23.6 to 21.5 MPa and from 3.2 to 1.9%, respectively) by increasing the PO fiber content from 0 to 30 wt %. Interestingly, the impact resistance of the composites increased with the increasing of the PO content: the Charpy’s impact energy increased from 3.6 (without fiber) to 4.4 kJ/m^2^ for the composite with 30 wt %. The results of the aerobic biodegradation under simulated marine conditions showed that the presence of PO fibers favored the physical disintegration of the composite increasing the biodegradation rate of the polymeric matrix: after 216 days, the composite with 20 wt % PO fibers showed a biodegradability of about 30% compared to 20% of the composite without fibers. Under real marine conditions, the specimens containing PO fibers showed higher weight losses and deterioration of tensile properties compared to those without fibers. Presumably, biodegradation occurred after colonization of the specimen, and the specimens with 20 wt % PO fibers showed well-developed biofilm consisting of bacteria and fungi on the surface after only 3 months of incubation in marine sediments, unlike the no-fiber specimens. Consequently, the persistence of an adequate mechanical performance for a relatively long period (1 year), due to a moderate rate of biodegradation in the marine environment, make the developed PHBV/PO composites particularly suitable for the production of relatively low-cost and biodegradable items which are usable in the sea and/or sand dunes, increasing the market opportunities for biopolymers such as PHBV and, at the same time, finding an eco-sustainable valorization for the PO fibrous residues accumulated in large quantities on Mediterranean beaches, which represents a problem for coastal municipalities.

## 1. Introduction

The world petro-plastic production reached about 335 million tons (Mt) in 2016 and is expected to double again over the next 20 years [1]. The United Nations Environment Program (UNEP) showed that, in 2015, 40% of plastic waste went to landfills and 14% was recycled, but 32% entered the ocean and accumulated as debris, representing a global threat to marine ecosystems [2]. Globally, 5 to 13 Mt of plastics end up in the oceans every year [3]. To reduce the problem of recycling petrochemical-based plastics and to minimize the environmental impact associated with their accidental deposition in marine habitats, increasing research efforts are being made in the field of bioplastics both by public and private institutions around the world. The biodegradability of bio-based materials in different natural environments, not only in composting systems, is an important property that makes their life-cycle more eco-sustainable compared to conventional petrol-based plastics. In many countries, traditional plastics are already being replaced with biodegradable ones; for example, in applications for packaging and bio-shoppers, even if their effects on marine organisms need to better evaluated [4,5]. Polyhydroxyalkanoates (PHAs) are a large family of polyesters, obtained by a wide range of bacteria cultivated under stressful conditions, with similar thermoplastic properties to conventional plastics [6,7,8,9]. Copolymers of hydroxybutyrate and hydroxyvalerate, including poly(β-hydroxybutyrate-co-β-hydroxyvalerate, PHBV), have thermoplastic properties similar to polypropylene (PP), good mechanical properties and are commercially marketed. PHAs also have excellent biodegradability: many aerobic and anaerobic microorganisms (bacteria, cyanobacteria and fungi) may degrade PHAs in several environments—in soil, in industrial/domestic compost, in fresh water and in various marine ecosystems [7,10,11,12,13,14]. Several factors influence PHA degradation rate, such as weather conditions, microbial population, temperature, pH, exposed surface area and so on [10]. Despite the numerous studies conducted on the mechanisms of biodegradation of PHBV in seawater, the majority of these studies have focused on very thin film specimens (a few µm thick) and the lifetime of PHBV-based products in marine environment is not clearly established. Given the ability to degrade in different marine environments, PHAs are among the most promising polymer candidates for the production of biodegradable items. However, their relatively high cost (7–12 €/kg) [7] compared to other biodegradable polymers such as poly-lactic acid (PLA) (2.5–3 €/kg), has somehow hindered research activity into their use in commodity applications such as packaging and service items, and restricted their use to high-value applications, such as those in medical and pharmaceutical sectors. Various efforts have been made to incorporate low-value materials such as starch [14,15,16,17] into PHBVs to reduce the cost of the final products. For the same purpose, the use of low-cost lignocellulose fibers coming from agricultural and industrial crops and the use of highly-available and low-cost natural fillers can allow the production of PHA-based composites more economically suited for commodities and service uses. Different lignocellulose fibers and natural fibers, such as kenaf, eucalyptus, rice straw, bagasse, cotton, sawdust, etc., have been tested [18,19,20,21,22] as fillers for thermoplastic polymeric matrices in order to lower the cost of the final product. In some cases, the developed composites showed promising mechanical and physical properties. Recently, the fibrous wastes derived from a seagrass, *Posidonia oceanica* (PO), have also been used as filler in the production of “green” composites [23,24,25,26,27], including PHA-based composites [28]. *Posidonia oceanica* is a Mediterranean endemic species that covers 60% of the seabed from 0 to 40 m depth [29]. The chemical composition of *Posidonia oceanica* fibers is similar to that of lignocellulosic materials from terrestrial plants consisting mainly of cellulose, hemicellulose and lignin. Large amounts of this material in the form of balls (*egagropili*) as well as whole plant fragments are deposited along many coastal beaches after storms. Stranded *Posidonia oceanica* residues play an important ecological role for the protection of coasts from erosion [30], but the presence of such biomass can represent a complex problem for coastal municipalities due to bad odors deriving from their decomposition, negative visual impact and high costs for their management and disposal. Every summer, many Italian coastal municipalities are forced to remove seagrass residues and dispose of them in landfills as municipal waste, with high costs. Italian legislation (Legislative Decree no. 75, 29 April 2010) allowed the use of these *Posidonia oceanica* wastes in compost production, up to a maximum of 20 wt % by fresh weight of the composting mixture. However, the high content of sand and salinity represent the main obstacles to the large use of this biomass for composting. Thus, the search for sustainable and cost-competitive alternatives to landfilling or composting is strongly encouraged.

In the present work, composites based on poly(3-hydroxybutyrate-3-hydroxyvalerate) and fibers of *Posidonia oceanica* were produced by melt mixing, processed by injection extrusion and characterized in terms of processability, rheological properties, thermal stability and mechanical performance. In order to improve the processability and the ductility of PHBV the addition of a plasticizer was necessary. For this reason, acetyl-tri-n-butyl citrate (ATBC) was used, being one of the most efficient plasticizers proposed for poly(hydroxybutyrate) (PHB) and its blends with PLA [31,32,33,34]. ATBC is non-toxic [35], accepted for contact with food [36] and is derived from naturally occurring citric acid. In view of the potential uses of the developed composites in specialized applications in marine environments, including marine restoration interventions, biodegradation/degradation tests were carried out in simulated and natural marine conditions in order to evaluate the effect of the presence of PO fibers on the aerobic biodegradation rate of the composites and on the lifetime of composite specimens (with thicknesses of 1.5–2 mm), respectively. In particular, the aerobic biodegradability of the composites was assessed at lab scale under simulated marine conditions by measuring the microbial carbon dioxide evolution from bioreactors. The degree of degradation of the composite specimens was evaluated in an outdoor marine culture system monitoring their weight loss and changes of morphology and mechanical properties over a period of one year.

## 2. Materials and Methods

### 2.1. Materials

The thermoplastic matrix used in this work is PHI002™ supplied in pellets from Naturplast^®^ (Caen, France). It is a poly(hydroxybutyrate-co-hydroxyvalerate) (PHBV), with 5 wt % valerate content. PHBV is characterized by a melting point of 145–150 °C, a density of 1.25 g/cm^3^, and a melt flow index of 10–20 g/10 min (190 °C, 2.16 kg). *Posidonia oceanica* (PO) balls (*egagropili*) were collected on a beach of the Ligurian Sea (Rosignano Solvay, Italy). They were washed with tap water to eliminate sand and other contaminants and then dried at 50 °C in an electric oven for 24 h. The ensuing material was milled using a lab-scale mill and sieved with a 500 µm sieve. The final length of the fibers was in a 1.5–2 mm range and the aspect ratio was in the 7–10 range. ATBC from Sigma Aldrich (Sigma Aldrich, St. Louis, MS, USA) was selected as plasticizer for PHBV. In addition, it is a bio-based, biodegradable and an almost colorless/odorless oily liquid. Calcium carbonate OMYACARB^®^2 provided by OMNYA^®^ (Oftringen, Switzerland) with fine grain size distribution (12 μm) was used as filler in order to reduce the cost of the final product and facilitate the removal of the product from the mold.

### 2.2. Composite Preparation

Composites containing 10, 20, and 30 wt % PO fibers, with respect to the total weight, were produced using 95/5 *w*/*w* PHBV/CaCO_3_ as polymeric matrix and 10 wt % ATBC on the total weight. The nomenclature of the samples was as follows: PCA for the composite without PO fibers (PHBV/CaCO_3_/ATBC) and PCA10, PCA20 and PCA30 for the composites containing the different fiber loadings: 10, 20 and 30 wt % of PO fibers, on the total composite weight, respectively. The composites were prepared by mixing the different components and then the ensuing mixtures were processed in a Thermo Scientific Haake Minilab Micro-compounder (Minilab), a co-rotating conical twin-screw extruder. The extruder operating conditions adopted for all the formulations are reported in Table 1. During the melting process in Minilab, the torque momentum and the pressure were recorded to evaluate the fiber effect on the melt fluidity.

The extruded composite filaments were cut to obtain pellets used for the subsequent thermal characterization and the lab-scale aerobic biodegradation test in seawater. For each formulation, specimens for the tensile test (Haake III type dog-bone tensile bars: width 10 mm, width in the narrow section 4.8 mm, thickness 1.35 mm, length 90 mm) and for the Charpy’s impact test (bars 80 × 10 × 4 mm) were produced by feeding the molten material directly from the Minilab to a Thermo Scientific HAAKE MiniJet II (Karlsruhe, Germany). Dog-bone specimens were also used for the degradation test in natural marine mesocosms.

### 2.3. Composite Characterization

Thermal properties of the starting materials and PHBV/PO composites in the form of pellets were evaluated by thermogravimetric analysis (TGA). Thermogravimetric measurements were carried out on about 20 mg of sample using a Q500 TGA (TA Instruments; New Castle, DE, USA), under nitrogen flow (100 mL/min), at a heating speed of 10 °C/min from room temperature to 600 °C. In addition, the isothermal stability of the raw materials at the processing conditions (170 °C) under nitrogen for 5 min has been also evaluated using an isothermal mode. The TGA measurements were carried out in duplicate. Tensile tests were performed on the injection molded Haake Type 3 (557–2290) dog-bone tensile bars of composites in accordance with ASTM D 638. Stress–strain tests were carried out at room temperature, at a crosshead speed of 10 mm/min, by an Instron 5500R universal testing machine (Canton, MA, USA) equipped with a 10 kN load cell and interfaced with a computer running the Testworks 4.0 software (MTS Systems Corporation, Eden Prairie, MN, USA). Impact test was carried out on V-notched specimens using a 15 J Charpy pendulum (CEAST 9050, Instron, Canton, MA, USA) following the standard method ISO 179:1993. In the Charpy test, the load is provided by the impact of a weight at the end of a pendulum. A crack starts at the tip of the V-notch and runs through the specimen. The material deforms at a strain rate of typically 10^3^ s^−1^. The energy which is dissipated during fracture is calculated from the height of the pendulum weight before and after impact. The dissipated energy is reported as the impact toughness per unit fracture cross-section (J/m^2^).

For each mechanical test, at least five replicates were tested for each sample. Morphological analysis was carried out by scanning electron microscopy (SEM), using a JEOL JSM-5600LV (JEOL, Tokyo, Japan) both on the cross-section of the dog-bone tensile specimens fractured in liquid nitrogen and on the cross-section of the specimens fractured after the tensile tests in order to evaluate the dispersion of the fibers into the polymeric matrix and the fiber/matrix interactions, respectively.

### 2.4. Biodegradation/Degradation Tests in Marine Environmental Conditions

In view of the possible application of the PHBV/PO composites in marine environments, aerobic biodegradation and degradation tests were performed under simulated marine conditions and in real marine mesocosms.

#### 2.4.1. Lab-Scale Aerobic Biodegradation Test

**Figure 1:**
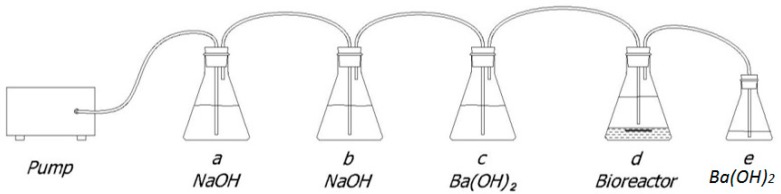
System set up for biodegradation test.

The aerobic biodegradation of the composites in simulated marine conditions was evaluated in accordance with a modified version of the ASTM D6691 method based on the measurement of the CO_2_ evolved from bioreactors containing the sample (pellets) placed at the seawater/marine sediment interface. The setup of the system used is shown in Figure 1.

The bioreactor contained 100 g of marine sediment (fine sand), 150 mL of sea-water and about 300 mg of composite sample (pellets). Seawater and the marine sandy sediment were collected in a coastal area near Rosignano Solvay (Tuscany, Italy), at 50 cm depth (43°21′ N, 10°26′ E). The test was conducted at room temperature in 300 mL flasks connected to each other with tubes where an air-flow rate of about 100–120 mL/min was fed through a pump. The flasks *a* and *b* contained 250 mL of a 5 M NaOH solution to remove CO_2_ from the air and the flask *c* 200 mL of a 0.04 M Ba(OH)_2_ solution, used to indicate the complete removal of CO_2_ from the air. The flask *e* contained 40 mL of a 0.1 M Ba(OH)_2_ solution to absorb the CO_2_ developed in the bioreactor during the degradation process. The amount of CO_2_ developed was calculated by the consumption of Ba(OH)_2_ in the flask *e*, determined by titration, carried out twice a week, with 0.1 M HCl using phenolphthalein as indicator. The amount of CO_2_ developed in each bioreactor was calculated according to the following equation:

mg_CO2_ = (V_(BaOH)2_·C_(BaOH)2_ − V_HCl_·C_HCl_)·44

where mg_CO2_ is the CO_2_ evolved from the bioreactor, V_Ba(OH)2_ is the volume in L of Ba(OH)_2_ (40 mL) in flask *e* at the beginning of the run, V_HCl_ is the volume in L of HCl needed for titration of Ba(OH)_2_ at the end of run, C_Ba(OH)2_ and C_HCl_ are the concentration of Ba(OH)_2_ and HCl, respectively, in mol/L, 44 is molecular weight of CO_2_. Filter cellulose paper (Whatman n.40) was used as positive control and flasks containing only seawater and sediment as blanks. The biodegradability of each sample was calculated as percentage (corrected for the blank flask emissions) of the overall theoretical amount of CO_2_ (ThCO_2_) that could be released, calculated on the basis of the initial organic carbon content of the sample, as shown below:

(1) $$Bio\;\%=\frac{\sum mgCO_{2}-\sum mgCO_{2,\;blank}}{ThCO_{2}}\times 100$$

Being an innovative natural fiber for composites production, and in order to evaluate the effect of fiber on composite degradation, the test in marine water was carried out even on PO fibers. For each type of sample, the test was quadrupled and the mean values were considered.

#### 2.4.2. Degradation Test in Marine Mesocosms

The changes in the weight and in the mechanical properties of dog-bone tensile specimens were evaluated over the time in an outdoor marine culture system, located at the Mariculture Center of Rosignano Solvay (Livorno, Italy) (43°22′ N, 10°26′ E). The system consisted of tanks (7000 m^3^) with continuous natural seawater flow equipped following a protocol, previously established for successfully growing seagrasses [37]. The water temperature in the tanks varied between 12 °C in winter and 27 °C in summer according to local seasonal variations of sea surface temperature on the littoral coast of Rosignano. Seawater pH ranged from 8 to 8.2 and the average salinity was 38. The composite PCA20 was selected for this test since it represented a good compromise between processability and mechanical performance, as reported in the Results and Discussion sections, and the PCA composite without PO fibers was used as comparison sample. At the beginning of the experiment (June 2016), the specimens were individually inserted into a nylon mesh bag (mesh size 1.5 mm). The bags were randomly placed into commercial plastic pots (30 cm × 15 cm × 10 cm) filled with natural marine sediment (fine sand) collected at a depth of 0.30 m along the coast near the Centre. The bags were covered with a layer of sediment (5 cm) and the pots were placed in the tanks at 50 cm depth. For each sample type, three specimens were collected at random at intervals of two–three months over the experimental period. Collected samples were washed with sterile seawater to remove the biofilm, dried overnight at 35 °C and weighted using a high-precision balance. The weight loss of collected specimens was determined as difference between the weight at sampling and their initial weight and expressed in percentage. All the collected specimens were tested for tensile properties in order to evaluate the changes of their mechanical properties over the time.

The resultant sample weight losses as well as changes in the tensile properties were considered indicators of PHBV degradation. In addition, after the first 3 months of incubation, specimens were collected and observed using both a Wild M3C stereo microscope (Leica, Wetzlar, Germany) and a scanning electron microscope (JEOL JSM-5410, Tokyo, Japan) to check for the formation of biofilm and the presence of degradation signs and microorganisms on their surface. For the SEM analysis, the samples were fixed in 2% OsO_4_ distilled water, dehydrated in ethanol and, after critical point drying, coated with gold.

## 3. Results and Discussion

### 3.1. Composite Processing and Characterization

**Figure 2:**
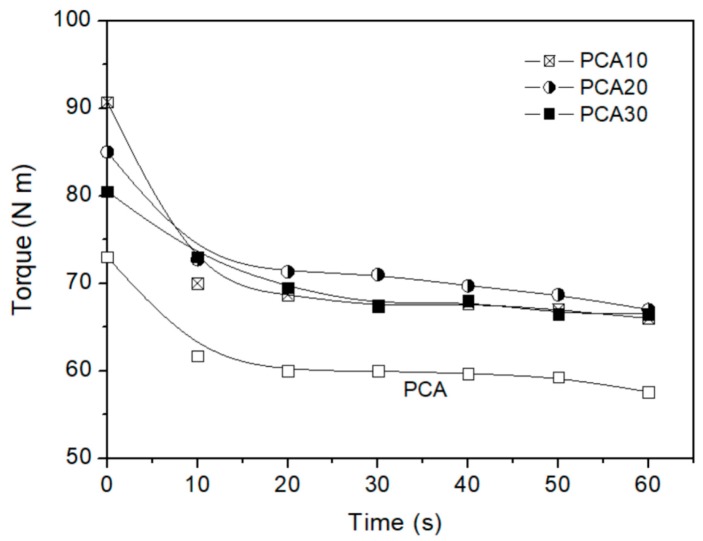
Torque vs time at 170 °C and rotor speed 100 rpm of the PCA (composite without fibers) and poly(hydroxybutyrate-co-hydroxyvalerate) (PHBV)/*Posidonia oceanica* (PO) composites.

In the processing of a polymeric composite, it is important to know how much energy is required to bring the mixture from an initial state to a homogeneous melt and how much time this process requires. The processing behavior of the investigated composites was quantified by the torque at the different mixing times and the end of mixing. The average torque–time curves obtained at 170 °C and a rotor speed 100 rpm for the PCA and PHBV/PO composites are reported in Figure 2.

The curves give indications on the mixing behavior and on the energetic effects of the process. The incorporation of the PO fibers in the PHBV based polymeric matrix increased the torque and consequently the energy required for the melt mixing. All the PHBV/PO composites showed the same final torque value of about 66.5 Nm: this can be attributed to the fact that, going from PCA10 to PCA30, the ratio *w*/*w* of ATBC/ PHBV increased from 0.13 to 0.175 compensating for the effect of the fiber content increase.

**Figure 3:**
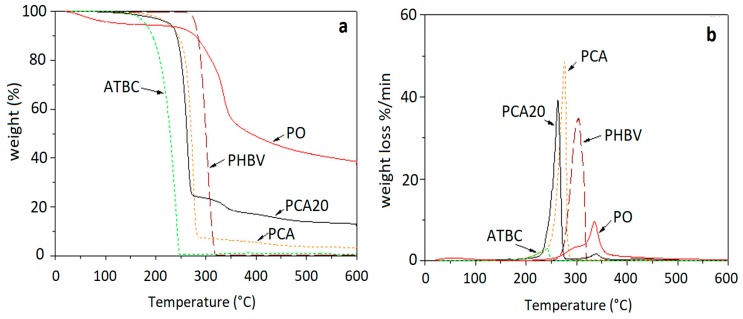
(**a**) Thermogravimetric (TG) and (**b**) derivate TG (DTG) curves of PO fibers, PHBV, acetyl-tri-n-butyl citrate (ATBC) and the developed composites.

The thermal stability of PO fibers, PHBV, ATBC and the developed composites in terms of thermogravimetric (TG) and their derivative (DTG) curves is shown in Figure 3. PO fibers show an initial limited weight loss (about 4%) up to 100 °C attributable to the residual humidity, then a weight loss of 50% is recorded from 250 to 450 °C corresponding to the main thermal degradation process. The broad peak of degradation of PO fibers is attributed to the overlapping of the degradation steps of the different components (hemicellulose, cellulose and lignin) of the fibers [26]. The onset temperature higher than 250 °C attests to the suitability of these natural fibers to be processed with thermoplastic polymer matrices, such as PHBV, without incurring thermal degradation. For PHBV, the degradation onset temperature was about 260 °C and the maximum rate decomposition temperature was 305 °C. No residue was recorded above 350 °C for PHBV. For all of the produced composites the thermal degradation started over 200 °C, with the main degradation peak over 250 °C.

**Figure 4:**
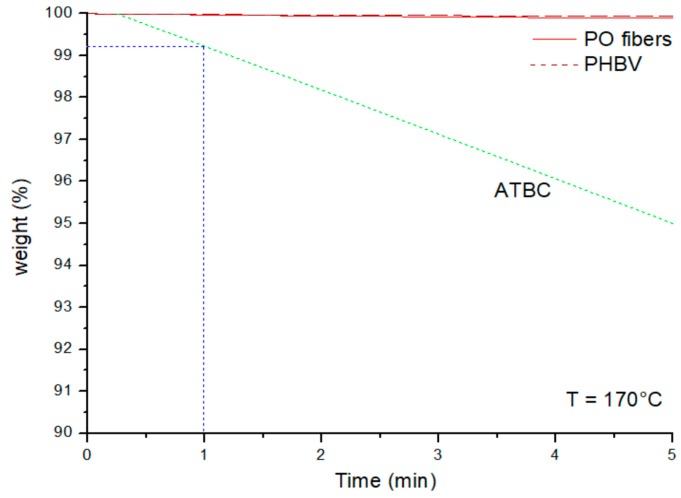
TG isothermal curves of the raw materials at 170 °C.

The thermal stability of the PHBV, PO fibers and ATBC at the composite processing temperature (170 °C) is shown in Figure 4. As expected, PHBV and dried PO fibers did not show significant weight losses at this temperature after 1 min, which is the typical maximum processing time of blends in an industrial extruder; ATBC showed a small weight loss, below 1%, after 1 min at 170 °C due to evaporation. These results are in accordance with those reported by Arrieta al. [38] for PHB and ATBC at 180 °C.

The mechanical properties of the composites, evaluated by tensile and Charpy’s impact tests, are reported in Table 2.

As shown, the Young’s modulus slightly increased and the elongation at break significantly decreased with increasing fiber loading, due to the stiffening effect induced by lignocellulosic fillers [39], while the stress at break remained almost constant at about 23.5 MPa for up to 20 wt % of fibers and reduced to around 21 MPa, increasing the fiber load to 30 wt %. As shown, up to 20 wt % of PO fibers (PCA20), the effect of the fibers resulted in being compensated by the increase of the weight plasticizer/PHBV ratio going from the PCA (ATBC/PHBV = 0.11) to the PCA20 (0.15); while for PCA30 (with 30 wt % PO fibers), the high presence of fibers led to an inevitable reduction of the tensile properties despite the higher ATBC/PHBV ratio (0.175). These results are in accordance with those of other authors [24,27,28] relating to composites containing *Posidonia oceanica* fibers. The observed behavior is typical of particle-filled polymeric matrices with poor or no compatibility between the components, meaning that stress transfer phenomena cannot occur and the filler particle becomes a stress concentrator leading to brittle fracture [40,41].

**Figure 5:**
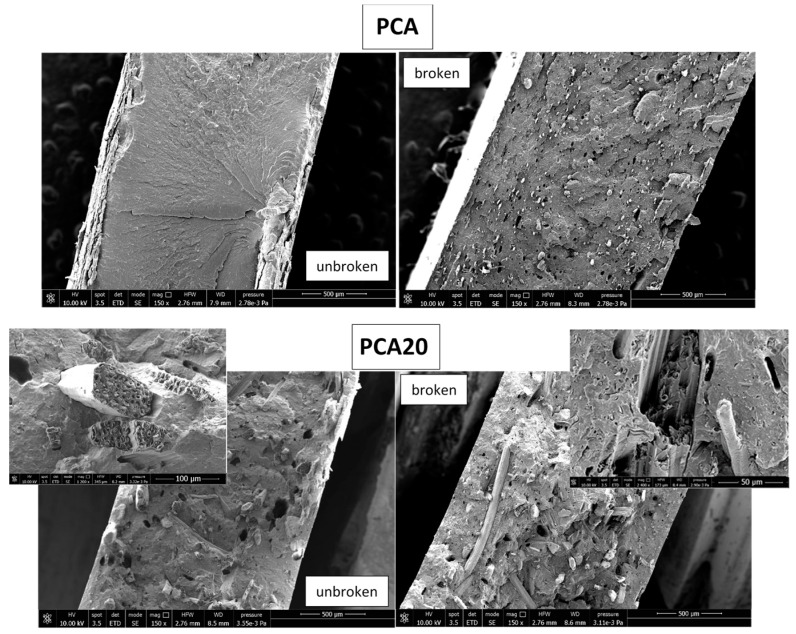
SEM images of the cross-sections of the PCA and PCA20 specimens before (unbroken samples) and after tensile tests (broken samples).

In order to better analyze the tensile results, SEM analysis was performed on the cross-section of the specimens before the tensile test and on the fractured section after tensile test. As example, the SEM images of the PCA and PCA20 specimens are reported in Figure 5.

As shown, in the unbroken PC20 specimen, the PO fibers are quite homogeneously distributed within the thermoplastic matrix. The interfacial interactions between the fibers and the matrix were not sufficiently strong to maintain the cohesion during the tensile tests, as shown in the broken specimen section where a significant fiber pullout is evident. The same results were observed also for the samples PCA10 and PCA30, which are not shown here for brevity.

As shown in Table 2, the presence of PO fibers slightly improved the capacity of the composite to absorb and dissipate impact energy compared to the polymeric matrix with no fiber. Impact resistance increase can be attributed to the poor matrix/fiber interaction. In fact, the impact failure of a composite occurs by factors such as fiber/matrix de-bonding, fiber and/or matrix fracture and fiber pull out. Fiber fracture dissipates less energy compared to fiber pullout, and this is common in composites with strong interfacial bonds, while the occurrence of the latter is a sign of a weak fiber/matrix bond [41,42,43]. The applied load transferred by shear force to fibers may exceed the fiber/matrix interfacial bond strength and the composites fracture in a brittle mode, which is in agreement with the experimental results.

### 3.2. Lab-Scale Biodegradation Test

**Figure 6:**
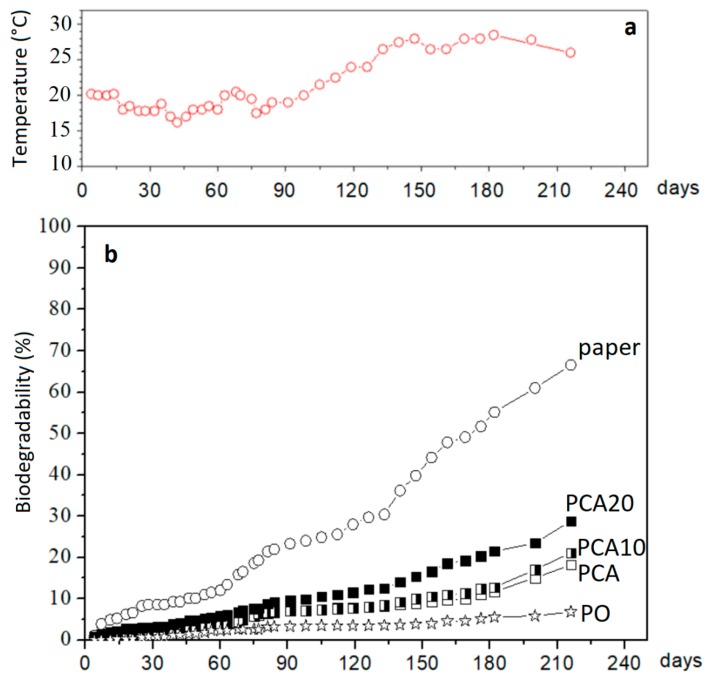
(**a**) Temperature measured during the lab-scale biodegradation test; (**b**) biodegradation curves under simulated marine environmental conditions.

The aerobic biodegradation curves obtained from the lab-scale test under simulated marine environmental conditions and the temperature recorded during the test are reported in Figure 6.

**Figure 7:**
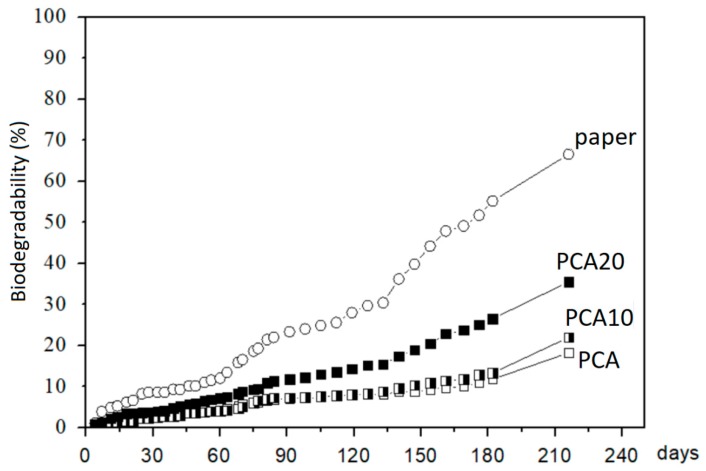
Biodegradability curves evaluated not considering the contribution of the PO fibers in the ThCO_2_.

At the end of the test (216 days), the biodegradation of the filter paper was about 70%, while the composite PCA20 reached a value of about 30% higher than that without PO fiber (PCA) and still growing. Therefore, the “relative biodegradation”, i.e., the biodegradation of the test material relative to the positive benchmark (paper, in this case), was about 40%. Considering the slow degradation rate of lignocellulosic PO fibers in natural environments that was confirmed also by this test (as shown in Figure 5), the percentage of biodegradation was recalculated using Equation (1), with the overall theoretical amount of CO_2_ (ThCO_2_) based only on the carbon content of PHBV and ATBC. In this way, the effect of the PO fibers on the biodegradation rate of the polymeric matrix was more evident, as shown in Figure 7. The biodegradation of the polymeric matrix increased with increasing PO fiber content, reaching a biodegradability value of about 35% in the case of composite PCA20 and 22% for PCA10, corresponding to a relative biodegradability of about 50 and 31%, respectively, showing that the PO fibers accelerated the biodegradation process.

In addition, it is widely known that plasticizers, including ATBC, speed up the disintegration phenomena of polymeric matrices due to the increasing polymer chain mobility as observed by Arrieta et al. [33] for PLA/PHB-ATBC blends under composting conditions. Consequently, and also in this case, in the developed composites, ATBC may have contributed to accelerating, together with the PO fibers, the disintegration of the specimens and accelerating, consequently, the biodegradation of the PHBV.

### 3.3. Degradation Test in Marine Mesocosms

**Figure 8:**
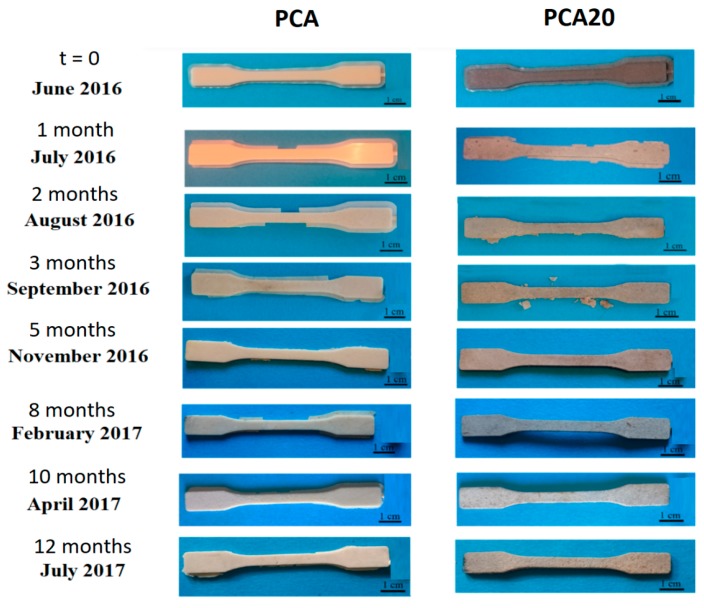
Photos of the tensile specimens at the start of the experiment (*t* = 0) and during the degradation in marine sediments.

The degradation test carried out on tensile specimens placed in marine mesocosms provided useful information regarding the degradation rate of the material in real marine environments. Photos of the specimens of PCA and PCA20, withdrawn after 1, 2, 3, 5, 8, 10, 12 incubation months are reported in Figure 8.

**Figure 9:**
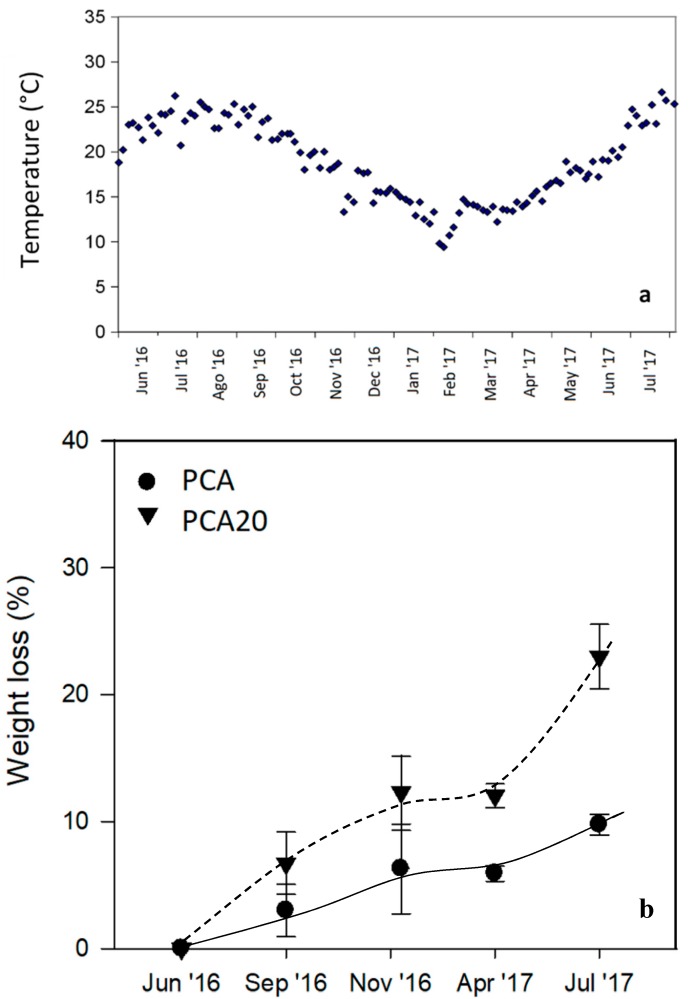
(**a**) Temperature measured during the degradation test and (**b**) weight loss of specimens over time in marine mesocosms.

**Figure 10:**
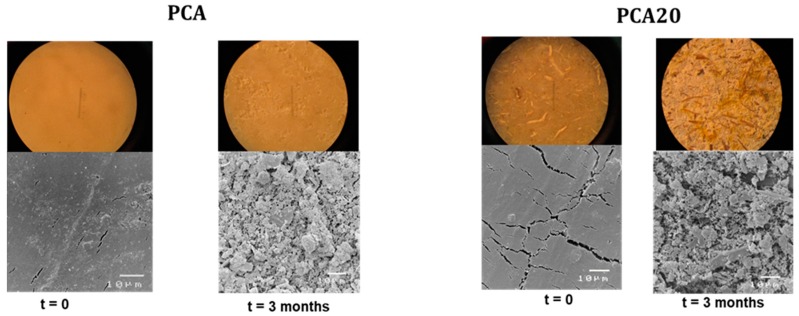
Images of PCA and PCA20 samples at stereomicroscope (above) and scanning electron micrographs (below) at the start of the experiment (time 0) and after 3 months of incubation in marine sediments.

As shown, the samples remained intact during the experimental period. Figure 9 shows the seawater temperature recorded during the test and the evolution of weight loss as a function of the incubation time. As shown, temperature ranged from about 20–25 °C in summer months and from 8 to 15 °C in winter months. For both samples, higher weight loss rates were observed in the summer months, especially for the PCA20 sample. These results are in accordance with the data in reference [44] that monitored the time-dependent changes in weight loss and mechanical strength of PHBV-based films, plates and fibers in seawater, showing that the degradation process was markedly dependent upon the temperature of the seawater. Besides this, PCA 20 was more readily degradable than PCA (composite without PO fibers) reaching an average weight loss of 23% after one year of immersion compared to a value of 10% of the PCA. This last value is similar to the percentage of weight loss of 12% reported by Imam et al. [14] for PHBV-based specimens with thickness of 2.5 mm after a year in natural seawater. After 3 months of incubation in marine sediment, stereomicroscopy revealed the presence of degradation signs on the surface of both the samples (Figure 10).

**Figure 11:**
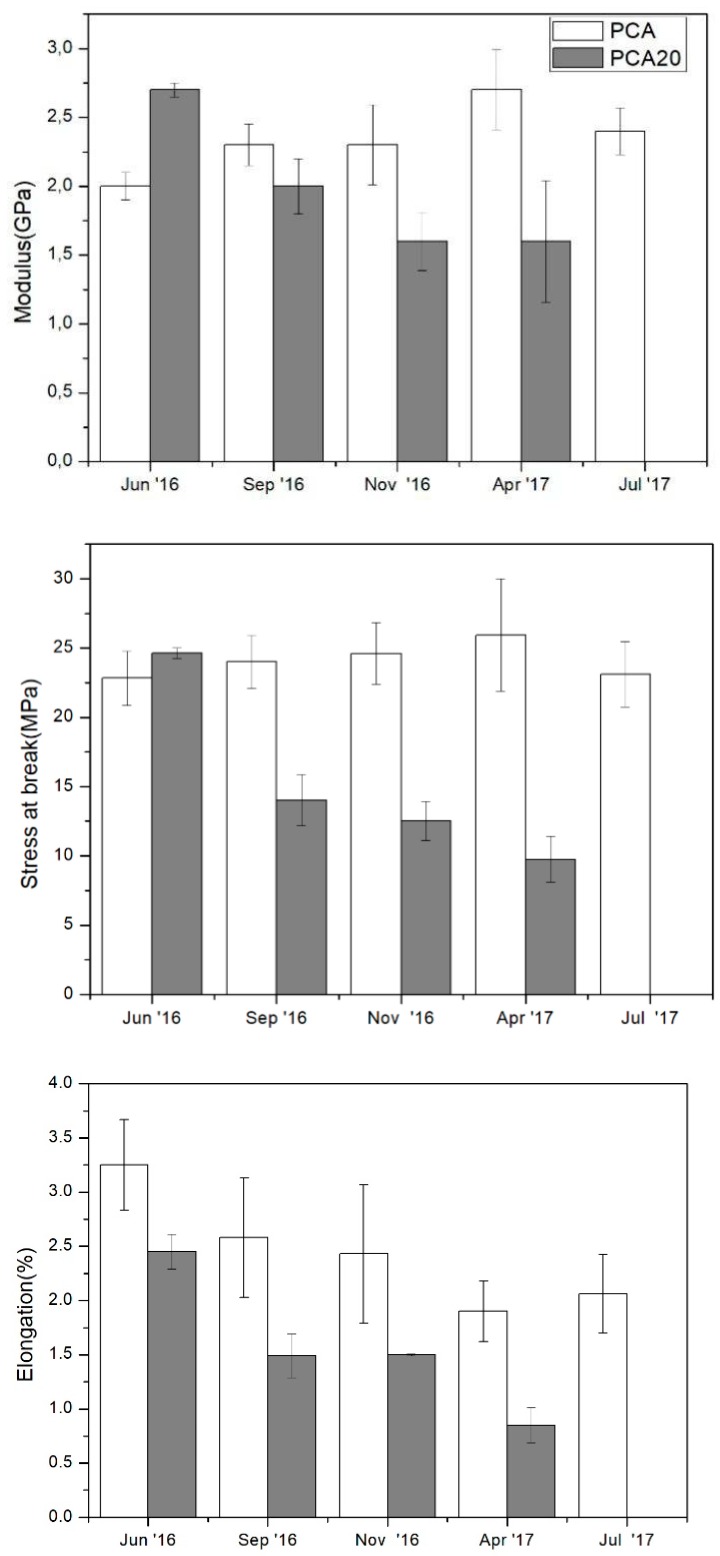
Tensile properties of PCA and PCA20 specimens after different periods of incubation in marine sediments in mesocosms.

SEM analysis confirmed the presence of the degradation signs, more evident in the sample with PO fibers (PCA20), and also showed a well-developed biofilm consisting of diatoms, bacteria and fungi on the PCA20 specimen surface. The Figure 11 shows the changes of the tensile properties of the PCA and PCA20 specimens collected from marine sediment over the incubation time. The results indicate that PCA specimens maintained their tensile properties almost constantly over time while PCA20 specimens showed a continuous decay of mechanical performance. The tensile strength at break and the elongation at break decreased of 60 and 68%, respectively, in 10 incubation months, and after 10 months of testing, it was not possible to evaluate their mechanical properties notwithstanding the fact that the PCA20 specimens were still recoverable, manageable and visually intact, as shown in the photos in Figure 8. This decay in the mechanical properties of the PCA20 with respect to the PCA is attributable to the presence of PO fibers that promoted the disintegration of the specimen, accelerating its degradation as revealed by the lab-scale biodegradation test. Thus, the persistence and maintenance of the mechanical properties for a relatively long period, due to a relatively slow rate of biodegradation in marine environments, make the developed composites particularly suitable for the production of biodegradable items usable in the sea and/or sand dunes. More importantly, it could be used to produce restoration tools, for example biodegradable pots for growing seagrass cuttings and seeds [45,46]. Currently, most of the transplanting systems used in restoration programs to anchor plants to the substrate are made with polyethylene and polypropylene [47].

## 4. Conclusions

Different amounts (10, 20 and 30 wt %) of *Posidonia oceanica* fibers were incorporated in a thermoplastic matrix based on PHBV and ATBC to produce melt processable and biodegradable composites for specialized applications in marine environments. The resultant composites were characterized in terms of processability, thermal/mechanical/morphological properties and biodegradability under simulated and real marine environmental conditions.

The processing of the composites at up to 30 wt % of PO fiber content did not create any difficulty, and the obtained composites showed good thermal stability and mechanical properties, especially in terms of stress at break and impact resistance. The biodegradation test results in the simulated marine environment showed that the presence of *Posidonia oceanica* fibers in the composites accelerated the biodegradation of polymeric matrix. After about 7 months in marine sediment, pellets with 20 wt % of PO fibers showed a biodegradability of 30%, corresponding to a relative biodegradability, with respect to cellulose, of 40%. The degradation test in marine mesocosm showed that after 3 incubation months in marine sediments, an evident microbial community consisting of bacteria and fungi colonized the surface of the composite specimens containing PO fibers. During the 12 months of testing, the specimens without fibers maintained their tensile properties almost constantly, while those with 20 wt % PO fibers showed a strong decay of their mechanical performance, and after 10 months it was not possible to determine them. These findings confirm that the presence of PO fibers, due the poor matrix/fiber adhesion, favored the physical degradation of the specimens accelerating the biological attack with a consequent decay of the mechanical properties, and that the degradation process is influenced by the seawater temperature: higher temperatures led to higher degradation rates.

On the basis of the results, it can be concluded that the production of composites based on PHBV and fibrous wastes of *Posidonia oceanica* is technically feasible by conventional extrusion and represents an eco-sustainable valorization of these residues which accumulate on sandy beaches, contributing to solving a real environmental and management problem. In addition, the maintenance of the mechanical properties for a relatively long period (a year) makes the developed composites particularly suitable for the production of biodegradable and relatively low-cost items usable in the seawater such as restoration tools and, after the disintegration of the polymeric matrix, the residual PO fibers would return to their native habitat.

## Figures and Tables

**Table 1:** Operating conditions used for the extrusion and injection molding process.

Extrusion Temperature (°C)	Screw Speed (rpm)	Cycle Time (s)	Injection Temperature (°C)	Injection Pressure (bar)	Molding Time (s)	Mold Temperature (°C)
170	100	60	170	210	15	60

**Table 2:** Mechanical properties of the composites with different PO fiber content.

Sample	Young’s Modulus (GPa)	Stress at Break (MPa)	Elongation (%)	Charpy’s Impact Energy (kJ/m^2^)
PCA	2.01 ± 0.10	23.60 ± 1.97	3.25 ± 0.42	3.61 ± 0.36
PCA10	2.37 ± 0.18	23.42 ± 1.87	2.63 ± 0.17	3.83 ± 0.26
PCA20	2.72 ± 0.05	24.62 ± 0.39	2.45 ± 0.16	4.14 ± 0.52
PCA30	2.38 ± 0.15	21.45 ± 1.63	1.92 ± 0.16	4.37 ± 0.24

The values are the mean ± SD of at least five determinations.
